# A synthetic medium to simulate sugarcane molasses

**DOI:** 10.1186/s13068-018-1221-x

**Published:** 2018-08-11

**Authors:** Felipe Senne de Oliveira Lino, Thiago Olitta Basso, Morten Otto Alexander Sommer

**Affiliations:** 1 Novo Nordisk Foundation Center for Biosustainability, Technical University of Denmark, Kemitovert 220, 2800 Kongens Lyngby, Denmark; 2 Department of Chemical Engineering, Polytechnic School, University of São Paulo, Av. Professor Lineu Prestes, 580 São Paulo, Brazil

**Keywords:** Yeast fermentation, Synthetic molasses, Microbial physiology, Industrial strains, Strain fitness, Pairwise cultivation

## Abstract

**Background:**

Developing novel microbial cell factories requires careful testing of candidates under industrially relevant conditions. However, this frequently occurs late during the strain development process. The availability of laboratory media that simulate industrial-like conditions might improve cell factory development, as they allow for strain construction and testing in the laboratory under more relevant conditions. While sugarcane molasses is one of the most important substrates for the production of biofuels and other bioprocess-based commodities, there are no defined media that faithfully simulate it. In this study, we tested the performance of a new synthetic medium simulating sugarcane molasses.

**Results:**

Laboratory scale simulations of the Brazilian ethanol production process, using both sugarcane molasses and our synthetic molasses (SM), demonstrated good reproducibility of the fermentation performance, using yeast strains, PE-2 and Ethanol Red™. After 4 cycles of fermentation, the final ethanol yield (g_p_ g_s_^−1^) values for the SM ranged from 0.43 ± 0.01 to 0.44 ± 0.01 and from 0.40 ± 0.01 to 0.46 ± 0.01 for the molasses-based fermentations. The other fermentation parameters (i.e., biomass production, yeast viability, and glycerol and acetic acid yield) were also within similar value ranges for all the fermentations. Sequential pairwise competition experiments, comparing industrial and laboratory yeast strains, demonstrated the impact of the media on strain fitness. After two sequential cocultivations, the relative abundance of the laboratory yeast strain was 5-fold lower in the SM compared to the yeast extract-peptone-dextrose medium, highlighting the importance of the media composition on strain fitness.

**Conclusions:**

Simulating industrial conditions at laboratory scale is a key part of the efficient development of novel microbial cell factories. In this study, we have developed a synthetic medium that simulated industrial sugarcane molasses media. We found good agreement between the synthetic medium and the industrial media in terms of the physiological parameters of the industrial-like fermentations.

**Electronic supplementary material:**

The online version of this article (10.1186/s13068-018-1221-x) contains supplementary material, which is available to authorized users.

## Background

Molasses is an inexpensive renewable carbon source used in several industrial bioprocesses. Its main use is in the production of fuel ethanol [1], but molasses is also a substrate for the production of butanol [2], spirits [3], sorbitol [4], citric acid [5], lactic acid [6], succinic acid [7], fructo-oligosaccharides [8] and single cell protein [9].

Molasses is produced via the separation of sucrose crystals that follow the water evaporation from clarified juice (from sugarcane or beet) during the production of crystal sugar. The concentrated juice facilitates sucrose crystallization. Sucrose crystals are removed via centrifugation, and the remaining viscous liquid is molasses. Molasses can be further recycled in this process in order to maximize sugar production. As a rule of thumb, the higher the number of recycling steps the molasses is subjected to, the poorer is its quality as a raw material for fermentation [10].

Despite the widespread usage of molasses in different industrial bioprocesses, its efficient utilization by microbial cell factories should not be taken for granted. Indeed, molasses is quite inhibitory to several organisms due to its salinity, osmolarity [11] and the presence of toxic elements and fermentation inhibitors [12–18]. These factors, coupled with process-related stresses [19], product toxicity [20], temperature stresses [21], contaminating bacteria metabolism toxicity [22] and competition for nutrients [23], complicate the deployment of new industrial strains using molasses as a raw material [24].

The composition and final quality of molasses vary a great deal among batches, having different titers of nutrients (e.g., minerals, sucrose, glucose, fructose, vitamins, fatty acids, etc.) and toxic compounds (e.g., aluminum and sulfites and thermal sugar degradation compounds) [25]. Consequently, industrial microbial strains are selected for their robustness and higher fitness in the stressful conditions imposed throughout the industrial bioprocess, including those constraints imposed by the quality of the raw material used for the preparation of the fermentation media [10, 14, 25, 26]. This variability and the difficulty of obtaining industrial raw materials are hurdles that impact the reproducibility and the applicability of the results from different groups developing new strains and bioprocesses based on commodity substrates, such as molasses.

To simplify the workflow and increase the reproducibility of experiments, synthetic media are optimal for laboratory scale experiments and gene function/metabolic pathways elucidations. Unfortunately, the current commonly available media (e.g., YPD, LB, and MRS) poorly replicate the industrial reality of sugar cane molasses fermentation media [27]. A synthetic medium, with a composition similar to those found in industrial media, would close this open loop between laboratory and industrial data, improving strain development efforts [24]. A synthetic medium, with a known composition, also has the clear advantage of allowing researchers to study the discrete impact of certain nutrients in the microorganism’s metabolism in given relevant growth conditions [28–30] and to also study the influence of such given nutrients in process-related conditions (e.g., different feeding regimens), allowing for the improvement of the established industrial bioprocesses [31].

To address this issue, we developed a simple and chemically semidefined culture medium, termed synthetic molasses (SM), which closely resembles industrial sugarcane molasses fermentation media. With a focus on microbial physiology and using the Brazilian ethanol fermentation as a process model, we compared the fermentability of SM against Brazilian and Indian sugarcane molasses [32], and we characterized the microbial growth and fitness of different strains in these media.

## Methods

### Chemicals

All chemicals used were of reagent grade and were obtained from Sigma-Aldrich (St. Louis, Missouri, USA), unless stated otherwise. The enzymatic kits for sugar quantification (sucrose, glucose and fructose) were acquired from Megazyme (Bray, Co. Wiclow, Ireland). The Brazilian sugarcane molasses samples (Mol_1 and Mol_2) were kindly provided by Prof. Thiago Basso (University of São Paulo, São Paulo, Brazil) and originated from sugarcane ethanol mills in the State of São Paulo. The India sugarcane molasses samples (Mol_3) were kindly provided by EM Agriton BV (Noordwolde, Friesland, The Netherlands).

### Yeast strains and maintenance

The *S. cerevisiae* strains used were Ethanol Red™ from Fermentis (Lesaffre, Marcq-en-Barœul, France), PE-2 (kindly provided by Prof. Luiz Carlos Basso, from Escola Superior de Agricultura Luiz de Queiroz, University of São Paulo, Brazil) and a YFP-producing laboratory strain CEN.PK102-5B (MAT*a ura3*-*52 his3∆1 leu2*-*3/112 MAL2*-*8*^*c*^ *SUC2*). According to Fermentis, Ethanol Red™ is a specially selected strain developed for the ethanol industry. PE-2 is an industrial isolate widely used by the Brazilian bioethanol industry [17]. The CEN.PK102-5B strain used in this study was constructed as described elsewhere [33], with minor modifications. The strain carried a plasmid expressing only one fluorescent protein and two empty plasmids for curing the auxotrophic markers.

The stock cultures were prepared by growing cells in shake flasks containing YP medium (1% yeast extract, 2% bacteriological peptone), with an initial glucose concentration of 20 g L^−1^. After overnight growth at 30 °C and 200 rpm, 20% (final concentration, v/v) glycerol was added, and 1 mL aliquots were stored at − 80 °C. The inocula were prepared by growing the stock cultures in YPD media. The yeast inocula were grown statically, at 30 °C, for 24 h.

### Media and culture conditions

YPD was prepared according to the manufacturer’s recommendation. Sugarcane musts were prepared by diluting the sugarcane molasses in tap water to 20° Brix. After dilution, the musts were centrifuged (10,000×*g* for 15 min, at 4 °C) in order to remove the solid impurities and were autoclaved at 121 °C for 15 min. This previous centrifugation of all media is important in order to remove any potential solid precipitate prior to autoclaving.

The SM medium was adapted from elsewhere [29], with modifications [10, 26, 34, 40–42] (Table 1):

**Table 1:** Composition and nutrient concentration of SM. Concentrations in g l^−1^

Category	Nutrient	Concentration
Carbon sources	Sucrose	144
	Glucose	18
	Fructose	18
Nitrogen sources	Peptone^a^	4.9
	(NH_4_)_2_SO_4_	0.1
	(NH_4_)_2_HPO_4_·4H_2_O	1.42
Organic acids	Trans-aconitic acid	2
	l-malic acid	1
	Citric acid	0.01
Mineral salts	NaCl	0.5
	MgSO_4_·7H_2_O	1.002
	CaCl_2_·2H_2_O	0.06712
	KCl	0.012
	MnSO_4_·H_2_O	0.0004
	ZnSO_4_·7H_2_O	0.0004
	FeCl_3_·6H_2_O	0.017
	Na_2_MoO_4_·H_2_O	0.031
	KI	0.012
	CuSO_4_·5H_2_O	0.0004
	H_3_BO_3_	0.0005
Vitamins	Inositol	0.01
	Nicotinic acid	0.01
	Calcium pantothenate	0.001
	Biotin	0.00001
	Pyridoxine hydrochloride	0.00004
	Thiamine hydrochloride	0.00004
	Para-aminobenzoic acid	0.002
	Ergosterol	2 ml^b^

^a^Preferably plant-based peptones

^b^6%, w v^−1^, ergosterol in ethanol solution

The simulation of the Maillard reactions originating from the molasses production processes was achieved by preparing a concentrated stock sugar solution [l^−1^: sucrose (432 g); glucose (54 g); and fructose (54 g)]. In this stock solution, the most common amino acids normally found in sugarcane juice were added as follows [34]: (l^−1^) glutamine (57 g); aspartic acid (33 g); and asparagine (21.3 g). This solution was then autoclaved at 121 °C for 15 min (“liquids” program, 2 bar pressure, CertoClave Multicontrol. Certoclave, Traun, Austria) [35, 36]. The macro-nutrient (up to 0.1 g l^−1^) solution was prepared as a 5× concentrated stock solution and autoclaved at 121 °C for 15 min. The micro-nutrient solution (below 0.1 g l^−1^) was prepared as a 100× concentrated stock solution and was filter sterilized (0.22 µm). The solutions were later mixed and diluted in PBS (pH 7.4) to their final concentrations. When needed, the final pH was adjusted to *ca*. 6.0 with KOH. The total sugar content in all the media was quantified enzymatically, according to the manufacturer’s recommendation.

### SM fermentability

To obtain enough yeast biomass for the fermentation experiments and to adapt the cells to the molasses-based must, an YPD preculture (previously incubated overnight at 30 °C) was inoculated in propagation medium (10° Brix molasses medium, enriched with yeast extract 5 g l^−1^) and was fed daily with fresh media, up to a final volume of 3 l, over the course of 3 days [37]. The pre-inoculum was grown in 5 ml YPD. Later, it was transferred to a total volume of 0.5 l of diluted molasses. This was later fed with 1.5 l diluted molasses, and after overnight growth was fed again with more 1.5 l of the same medium.

The industrial ethanol fermentations were simulated in triplicate as described elsewhere [38]. The fermentations were carried out in 50-ml centrifuge tubes. In the first cycle, the cells from the propagation culture were added to each tube in an amount corresponding to 10% (w v^−1^) of the final volume. The cells were fed with the proper must and were incubated for 5 h at 30 °C without agitation and were left at room temperature overnight. The following day, the cells were separated from the fermentation wine by centrifugation (4996×*g*, 20 min at 4 °C), and wine from the previous cycle was added (70% wet weight w w^−1^) to simulate the industrial centrifuge efficiency. For the PE-2 fermentations, the biomass was adjusted for 3 g per tube (wet basis), after two cycles of fermentation, by removing the excess biomass using a clean spatula. The cells were further diluted and resuspended in demineralized water (30% wet weight w w^−1^) before the addition of 1 M sulfuric acid to a final pH of approximately 2.5. After the incubation in acid at room temperature for 1 h, feeding of fresh must was initiated, restarting the process.

The ethanol yield was calculated as described elsewhere [38]. For such, a correction factor for high cell density microbial cultivations was used [39]. Based on the previous published data, yeast cells have a volume of circa 0.7 ml g^−1^ (wet basis) containing an ethanol concentration equal to the cell-free supernatant [38]. In that way, ethanol produced was calculated as the mass balance difference between the ethanol content from the end of each fermentation cycle (accounting ethanol from the cell-free centrifuged wine plus the pelleted yeast biomass) and the ethanol present in the inoculum (returned wine and the pelleted yeast biomass from the previous cycle). The ethanol yield was calculated based on the total sugar supplied, as shown below (Eq. 1):

1 $${\text{Ethanol yield}}\, = \,K \, \times \,\{ \left( {{\text{Vw}}\, + \,0.7 \, \times \, P} \right) \, \times \, {\text{ET}}\, - \,\left( {{\text{Vrw}}\, + \,0.7 \, \times \, {\text{Pp}}} \right) \, \times \, {\text{ETp}}$$

$$K = \frac{10000}{{64.75\, \times \, Vs \, \times \, {\text{TRS}}}}$$

Vw is the volume (ml) of centrifuged wine; *P* is the pelleted yeast biomass (g); ET is the ethanol titre in centrifuged wine (%v/v); Vrw is the volume of returned wine from the previous cycle; Pp is the pelleted yeast biomass from the previous cycle (inoculum); ETp is the ethanol titer (%v/v) in centrifuged wine from the previous cycle (inoculum); Vs is the volume of substrate (ml); and TRS is the total reducing sugar of substrate (g 100 ml^−1^). Conversion factor 64.75 ml_ethanol_ 100 gTRS^−1^, equivalent to 51.11 g_ethanol_ 100 gTRS^−1^ [38].

The ethanol productivity was measured via the CO_2_ release, by weighting the tubes hourly. The viability was measured via flow-cytometry (BD LSRFortessa™, BD Biosciences, Franklin Lakes, New Jersey, USA), using propidium iodide dye as a viability marker, according to manufacturer’s recommendations.

The carbohydrate titer and composition (sucrose, glucose and fructose) from the fermentation media were inferred via an enzymatic analysis (K-SUFRG kit, Megazyme, Bray, Co. Wiclow, Ireland). The concentration of the fermentation metabolites (glycerol, ethanol, and acetic acid) was determined by high-performance liquid chromatography (HPLC) (UltiMate 3000, Thermo-Fischer Scientific, Waltham, Massachusetts, USA). The metabolites were separated using an Aminex HPX-87H ion exclusion column (Bio-Rad, Hercules, California, USA) and were isocratically eluted at 50 °C, with a flow rate of 0.6 ml min^−1^, using a 5 mM sulfuric acid solution as mobile phase. The detection was performed refractrometrically.

### Fitness of yeast strains in pairwise competition assays

The yeast strain fitness was analyzed via pairwise competition assays, where the laboratory strain was cocultured in the presence of one of the industrial yeast strains used: Ethanol Red™ or PE-2.

The molasses-based media were diluted for 20° Brix, and the SM was diluted 10×, using sterile demineralized water.

The biomasses of the inocula were estimated via optical density at 600 nm (OD_600_) and were adjusted to 1.0 by diluting the inocula in the proper growth media. This inoculum was later transferred to a microplate containing the same media, with a final OD_600_ of 0.1. The microplates were incubated at 30 °C under constant agitation (*‘fast’* double-orbital agitation mode), and the OD_600_ values were checked every 15 min for 24 h, using a microplate reader (Synergy H1™, Biotek Instruments Ltd, Winooski, Vermont, USA).

During these competition experiments, both the industrial yeast strains (PE-2 or Ethanol Red™) were cocultured in equal initial proportions (0.1 for each strain) compared with the laboratory strain CEN.PK102-5B, in the different media indicated above. During the cultivation, the OD_600_ values of the strains were measured every 15 min. After 24 h of cultivation, a new identical plate was prepared with fresh media, and each well was inoculated from its correspondent from the previous plate (100 µl inoculum in 200 µl fresh media). This new microplate was also incubated overnight under the same conditions.

A sample from each well (10 µl) was taken after the overnight cultivation and was transferred to a new microplate and diluted in 190 µl PBS buffer (pH 7.4) for the culture composition analysis. This was done via yeast cell identification (SSC versus FSC) and an YFP fluorescence (excitation at 510 nm, emission at 545 nm) measurement using flow cytometry (BD LSRFortessa™, BD Biosciences, Franklin Lakes, New Jersey, USA).

### Statistical analysis

The statistical analyses were performed using the software GraphPad Prism 7.04. For comparing the main fermentation parameters—ethanol yield and viability—among different media during each fermentation cycle, and the community structure in the pairwise competition experiments (for each analyzed time point), multiple t tests (statistical significance analysis with alpha value of 0.05) were performed.

## Results

### A synthetic medium with a similar composition to sugarcane molasses

Sugarcane molasses has both nutrients and compounds, which are potential inhibitors of microbial cells. To develop the SM medium, a basal molasses medium, described elsewhere [29], was modified based on the average sugarcane molasses composition described in other studies [10, 26, 34, 40–42], in order to obtain a final chemical composition similar to those observed in actual sugarcane molasses samples. An overview of all SM formulations developed throughout this study, and reasons for altering them are depicted in Additional file 1. The comparison between the average sugarcane molasses media (20° Brix) and the SM composition is depicted in Table 2.

**Table 2:** Chemical composition of the sugarcane molasses and SM media (in % w v^−1^)

Parameter	Sugarcane molasses^a^	SM
C/N^b^	57–209	180
N	0.13–0.5	0.1
P	0.01–0.05	0.05
K	0.6–1.8	0.001
Mg	0.01–0.04	0.01
Ca	0.02–0.06	0.002
Trans-aconitic acid	0.1–0.7	0.2
l-Malic acid	0.04–0.14	0.1
Citric acid	0.006–0.07	0.001

^a^The values were approximated for a sugarcane molasses-based media containing approximately 20° Brix

^b^C/N ratio, based on the ratio between the fermentable sugars and the total nitrogen content (w w^−1^)

In addition to sucrose, glucose and fructose were added in proportions similar to those observed in molasses, in which sucrose comprises approximately 80% of the total fermentable sugars and glucose and fructose make up the remaining 20%, in equal proportions [43]. The total nitrogen amount was adjusted in order to achieve a ratio between NH_4_^+^ and R-NH_2_ that was similar to those observed in sugarcane molasses [34]. The proportion between the total nitrogen and fermentable sugars (Carbon Nitrogen ratio; C:N) was also corrected [10, 44]. The most relevant organic acids found in sugarcane molasses—trans-aconitic acid and l-malic acid—were also added in relevant titers and proportions [34, 44]. Known toxic elements, such as aluminum, were not included in the SM composition, in view of their widespread variability in real molasses samples [34, 44]. The values of Ca, K, citric acid and C/N were based on the literature data [28] and left unchanged. Citric acid is not metabolized by *S. cerevisiae*, and can have an impact on its physiology only in considerably higher titers [45]. For such reasons, its value was left unchanged. This nutrient could have its value altered if another microorganism was employed.

It is important to notice that the quantified values of nutrients in the SM are related solely regarding those added from pure salts. Values of K and Ca from complex nutrient sources, such as peptones, may vary greatly [46]. We have decided to be conservative with these values in order to keep the final value within an acceptable threshold.

### SM reproduces the cell viability and fermentation kinetics of molasses-based fermentations with cell recycle

In Brazil, ethanol production from sugarcane raw materials makes use of a peculiar fermentation setup called the *Melle*-*Boinot* fermentation. A fed-batch fermentation with a very high yeast cell density is performed for several consecutive batches throughout the entire crop season (approximately 250 days). This process depends on the recovery of the yeast biomass (more than 90%) after each cycle of fermentation via the centrifugation of the final wine. After recovery, the yeast biomass is acid washed (pH 2.5 for 1 h) as a preventative method for controlling the contaminating lactic acid bacteria population that inhabits the process. After this acid wash procedure, the biomass is sent back to the fermenters, and a new fermentation cycle starts over [38]. Up to three fermentation batches are completed each day.

**Fig. 1:**
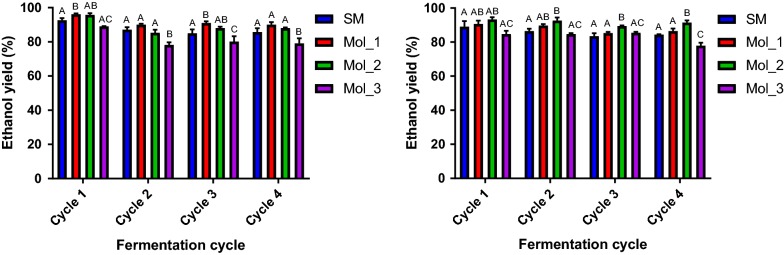
Fermentation performance of yeasts Ethanol Red™ and PE-2 in SM and molasses-based media. Comparison between the ethanol yield (in % of maximum theoretical yield) and ethanol titer (in g l^−1^). A Ethanol Red™ ethanol yield, B PE-2 ethanol yield, C Ethanol Red™ ethanol titer and D PE-2 ethanol titer. The fermentations were performed using the SM and molasses-based media (Mol_1, Mol_2 and Mol_3) throughout 4 consecutive cycles, simulating the Brazilian ethanol production process. The yeast biomass was kept within 10% (w/w; wet basis), and an acid wash (pH 2.5, 1 h incubation at room temperature) was performed before each new round of fermentation from the second cycle onwards. The tubes were incubated at 30 °C for 7 h and overnight at room temperature. The fermentations were performed in triplicate. For ethanol yield values letters indicate if averages are statistically similar (equal letters) or different (different letters)

The industrial strains PE-2 and Ethanol Red™ were propagated in supplemented diluted sugarcane molasses, in order to precondition the yeast population to this harsh substrate. The fermentations were performed with 20° Brix molasses (the total sugar concentration ranged from 131.4 to 188 g l^−1^, due to substrate variability) and the SM in its original formulation (sugar content of 180 g l^−1^), during 4 consecutive fermentation cycles, in triplicate. The composition of the media used (i.e., carbohydrate titer and composition, and organic acids (i.e., aconitic acid, lactic acid and acetic acid) concentration can be found in Additional file 2. The ethanol (fermentation) yield and the ethanol titer at the end of each fermentation cycle are presented below (Fig. 1).

Overall, the ethanol yield values were kept in a similar range, for both strains, throughout the fermentation cycles—i.e., a median value, for all fermentations, of 87.2 ± 3.9% for PE-2 and 87.6 ± 5.1% for Ethanol Red™. However, the yields varied substantially between the different media (ranging from approximately 78% in Mol_3 to 95% in Mol_2). This variability is most likely related to the molasses composition [10] and strain performance. For the Brazilian molasses (Mol_1 and Mol_2), the ethanol yield ranged from approximately 85–96%. Although this value range was greater than what is stated as the current norm for the industry (i.e., 90–92%) [17], it was in accordance with what is observed for laboratory scale molasses fermentations [38]. For Mol_3, both the final ethanol titer and yield were consistently lower (approx. 47 g l^−1^ and 82%, respectively) when compared against the SM and Mol_1 and Mol_2 (approx. 67 g l^−1^ and 87% for SM; 68–74 g l^−1^ and 85–96%, for Mol_1 and Mol_2, respectively). For the SM, Mol_1 and Mol_2, the final ethanol titers are within the range expected for such fermentations (i.e., 7–12% v v^−1^) [47]. The yield values also fell within the expected range (i.e., mean values ranged from 87.7 ± 3 to 91.9 ± 2.5%) [38].

**Fig. 2:**
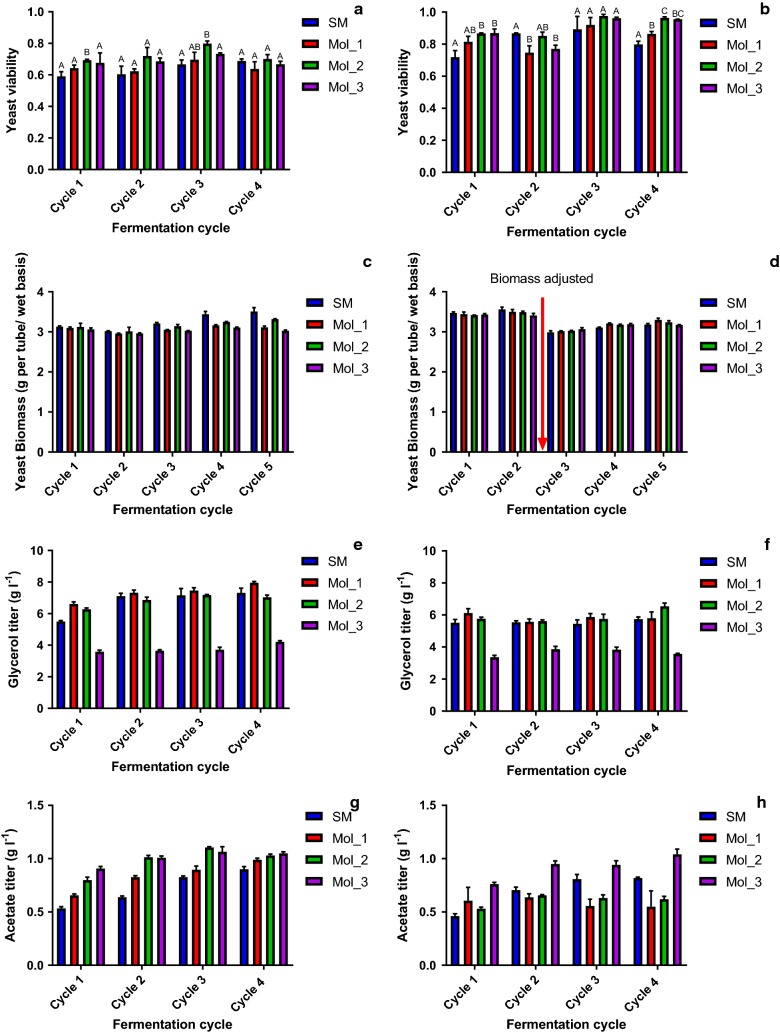
Key parameters of the fermentation with the SM and molasses (Mol_1, Mol_2 and Mol_3) media. **a** Ethanol Red™ viability values (% viable cells). **b** PE-2 viability values (% viable cells). **c** Ethanol Red™ biomass values (g, wet weight). **d** PE-2 biomass values (g, wet weight) biomass values (g, wet weight). **e** Ethanol Red™ glycerol titers (g l^−1^). **f** PE-2 glycerol titers (g l^−1^). **g** Ethanol Red™ acetate titers (g l^−1^). **h** PE-2 acetate titers (g l^−1^). The biomass was measured by weighing the pelleted biomass after centrifugation and removal of fermented wine. The viability was measured via flow-cytometry using propidium iodide as the viability marker. Glycerol and acetate were measured via HPLC. For viability values, letters indicate if averages are statistically similar (equal letters) or different (different letters)

The other relevant fermentation parameters analyzed (i.e., biomass, viability, glycerol and acetic acid titers) were also similar for the SM and the Brazilian industrial media Mol_1 and Mol_2, but the Indian media Mol_3 differed substantially (Fig. 2).

The biomass increase, measured as the yeast biomass gain along the cycles (Fig. 2a, b), was kept below 10% throughout all the fermentation cycles, in agreement with previous studies [48].

All the media supported growth and maintained the yeast throughout the fermentation cycles. Ethanol Red™ showed consistently lower viability values (i.e., ranging from 60 to 80%) compared to the PE-2 fermentations (i.e., values ranging from 71 to 97%). Although the viability levels observed in the Ethanol Red™ fermentations were low compared to those reported in the literature (i.e., 80 to > 90%) [17], they were in accordance with the values observed for this particular strain when using a sugarcane-based broth in sequential fermentation cycles [49]. This difference between the viability of both strains suggested that PE-2 had a higher fitness in this fermentation setup using sugarcane molasses-based media. For both strains, the SM provided intermediate viability values (i.e., ranging from 60% to 70% for Ethanol Red™, and from 71 to 90% for PE-2) compared to all three molasses. Both yeast strains were propagated in a diluted 10° Brix Mol_3 supplemented with 5 g l^−1^ of the yeast extract. Even though the viability in the Mol_3 fermentations was comparatively high for other media (i.e., 66% to 73% for Ethanol Red™ and 77–96% for PE-2), the viability during the propagation was lower than what was observed during fermentation (60% for PE-2).

The glycerol values ranged from 5.5 to 7.9 g l^−1^ for Ethanol Red™ and were 5.5–6.5 g l^−1^ for PE-2 (for SM, Mol_1 and Mol_2), which were within the expected ranges for this fermentation, where approximately 10% of the consumed C is converted into this metabolite [17, 38]. Again, Mol_3 had consistently lower glycerol titers (approx. 3.6–4.2 g l^−1^ for Ethanol Red™ and 3.4–3.8 g l^−1^ for PE-2). This lower value probably correlated with the lower carbohydrate concentration in this media compared to the others (approx. 131 g l^−1^ versus 165 to 188 g l^−1^), and this was also observed for the ethanol titer.

For the acetate levels, both strains showed similar values, ranging from approximately 0.5–1 g l^−1^, indicating a limited influence of the media or strain on the final acetate titers. Overall, the SM had lower acetate titers (e.g., 0.5 g l^−1^) during the initial cycles of fermentation, but at the final cycle, most of the values clustered between 0.55 and 1.0 g l^−1^ (PE-2) or between 0.9 and 1.0 g l^−1^ (Ethanol Red™).

When the product yield coefficients (ethanol yield—Yse; CO_2_ yield—Ysc; glycerol yield—Ysg; acetic acid yield—Ysa; g_product_ g_substrate_^−1^) were compared within the different broths, the SM broth yield values were similar to those observed in the actual industrial broths (Table 3).

**Table 3:** Main conversion yield coefficients (g_product_ g_substrate_^−1^) from the 4th fermentation cycle from the different broths

Strain	Broth	Yse	Ysc	Ysg	Ysa	g_glycerol_ g_ethanol_^−1^
Ethanol Red™	SM	0.44 ± 0.01	0.48 ± 0.00	0.04 ± 0.00	0.01 ± 0.00	0.09 ± 0.00
	Mol_1	0.46 ± 0.06	0.47 ± 0.00	0.04 ± 0.00	0.01 ± 0.00	0.09 ± 0.00
	Mol_2	0.45 ± 0.00	0.44 ± 0.00	0.04 ± 0.00	0.01 ± 0.00	0.09 ± 0.00
	Mol_3	0.40 ± 0.01	0.44 ± 0.16	0.03 ± 0.00	0.01 ± 0.00	0.08 ± 0.00
PE-2	SM	0.43 ± 0.00	0.47 ± 0.00	0.03 ± 0.00	0.01 ± 0.00	0.08 ± 0.00
	Mol_1	0.44 ± 0.01	0.45 ± 0.01	0.03 ± 0.00	0.00 ± 0.00	0.08 ± 0.01
	Mol_2	0.47 ± 0.01	0.48 ± 0.00	0.04 ± 0.00	0.00 ± 0.00	0.09 ± 0.00
	Mol_3	0.40 ± 0.01	0.44 ± 0.00	0.03 ± 0.00	0.01 ± 0.00	0.08 ± 0.00

*Yse* ethanol yield, *Ysc* CO_2_ yield, *Ysg* glycerol yield, *Ysa* acetic acid yield, *g*_*glycerol*_ *g*_*ethanol*_^*−1*^ ratio between the glycerol and ethanol produced (g g^−1^)

### Media composition impacts yeast fitness in pairwise cultivations

Cocultivation experiments are efficient methods for assaying the fitness of individual strains in a given mixed population [50]. In view of the nonaseptic nature of the process, a complex yeast community is found in industrial bioethanol fermentations [51]. To analyze the fitness of the strains in the molasses-based medium, we performed pairwise competition experiments with each of the two aforementioned industrial yeast strains against the laboratory strain CEN.PK102-5B.

The strains were inoculated, in equal proportions, in YPD, SM (10× diluted in sterile Milli-Q^®^ water), Mol_1, Mol_2 and Mol_3 (2° Brix molasses-based media) and were cultivated in microplates. The population structure was inferred via flow-cytometry, separating cells by their YFP fluorescence. After an overnight cultivation, a sample from each well was taken for the community structure analysis, and a new plate was inoculated from a cell suspension from the previous one. This procedure was repeated until the laboratory strain could not be identified in the cultivation media.

The community structure was assayed just after the inoculation in the first microplate [1st plate at time 0 h (*t*_0_)] and after the cultivation of the first and second microplates [1st plate, end of cultivation (*t*_f_), and 2nd plate, end of cultivation (*t*_f_)].

To exclude any possibility of the inhibition of strain CEN.PK102-5B by metabolites produced by Ethanol Red™ and PE-2, CEN.PK102-5B was also grown in all the aforementioned media (YPD; SM, Mol_1, Mol_2 and Mol_3), which was further diluted to 1× with either sterile Milli-Q^®^ H_2_O or with the supernatants of Ethanol Red™ and PE-2 and was grown in the same media. No indication of inhibition was observed (data not shown).

**Fig. 3:**
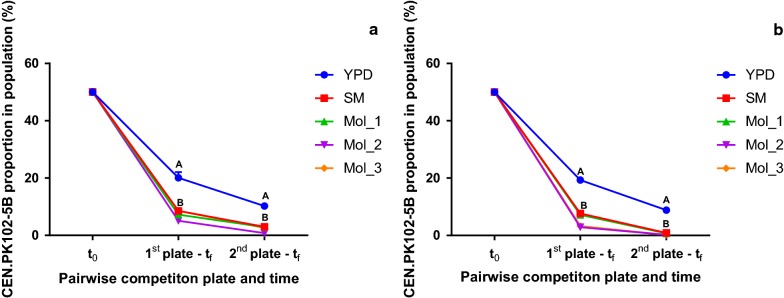
Synthetic and industrial molasses media favor the growth of industrial yeast strains in competitive fitness experiments. CEN.PK102-5B proportion (%) in the population during pairwise cultivations with Ethanol Red™ (**a**) and PE-2 (**b**) in YPD, SM and molasses-based media (Mol_1, Mol_2 and Mol_3). *t*_0_ = 1st plate at time 0 h; 1st plate − *t*_f _= the end of the cultivation of the first microplate; 2nd plate − *t*_f _= the end of the cultivation of the second microplate. Letters indicate if averages values from the proportion of CEN.PK102-5B in the community structure are statistically similar (equal letters) or different (different letters)

After two sequential cultivations, the CEN.PK102-5B population dropped from 50% to approximately 20% and then to approximately 10% of the total cells in YPD between the 1st plate t_f_ and the 2nd plate *t*_f_, demonstrating that the Ethanol Red™ and PE-2 grew faster compared to the lab strain, even at the optimal laboratory growth conditions (Fig. 3).

In the SM and in all the molasses-based media cultivations, CEN.PK102-5B was rapidly outcompeted by both industrial strains in the cocultivation experiments. However, the relative abundance of CEN.PK102-5B was significantly higher in the common laboratory medium YPD.

## Discussion

In this work, we designed a synthetic medium that had a similar composition to industrial sugarcane molasses media. Important aspects of the composition of molasses, such as its low nitrogen content, carbohydrate composition, carbon/nitrogen proportion, Mailard reaction products, salts and organic acid composition, were taken into consideration [1, 10, 34, 44, 52]. These new synthetic media were benchmarked against three different industrial molasses media, using a simulation of the Brazilian sugarcane ethanol production process as a case study. Its role as an important tool for industrial strain selection was also demonstrated, via pairwise cultivations with industrial and laboratory yeast strains, highlighting the influence of media composition over yeast fitness.

The Brazilian ethanol production process makes use of a cell recycling system. In this particular setup, some fermentation parameters are key for the process: ethanol yield and yeast viability, both of which are heavily influenced by the quality of the raw material [25]. For such parameters, a considerable variation was found (Figs. 1, 2), even within the different molasses media. SM was the medium that performed with most reproducibility and similarity to all media for both strains. Overall, ethanol yield and viability values obtained with SM fermentations were comparable to approximately 72% of all fermentations. Such comparability suggests that the physiological conditions found in most of fermentation batches and replicates (for all media and strains used) were being efficiently simulated by SM. Some variation was also observed among the parameters between both strains. This suggests that yeast strain also plays an important role in the reproducibility of data for laboratory scale fermentations [38]. PE-2 showed, consistently, a higher viability rate in all the media compared to Ethanol Red™. This is, most likely, an indication of the previous selection of PE-2 for sugarcane molasses-based fermentations, from where it was originally isolated [17].

Mol_3, obtained from Indian molasses, consistently yielded lower ethanol titer values and yields compared to the other media. The lower sugar content of Mol_3 suggests that Mol_3 was a less pure and more exhausted molasses, which may have passed through several cycles of crystallization compared to molasses B or C [10]. However, the difference may also reflect geographical differences between the Indian and Brazilian sugarcane molasses. Further tests are required to assess this.

Glycerol is constantly produced by yeast cells as a by-product under anaerobiosis to maintain the redox balance due to the NADH generation during cell growth and for coping against osmotic stresses [53]. A reduction in the glycerol:ethanol ratio may be related to the presence of inhibitors (e.g, organic acids and aluminum) in the broth [19], or by other stressful factors like high temperature, bacterial contamination and competition for nutrients against different yeast strains. We have not tested such conditions in this study. Futile cycles are energy sinks that demand yeast metabolism to divert more carbon towards catabolism, favoring this ratio towards ethanol production at the expense of biomass and glycerol production [54]. The reduced glycerol production on Mol_3 might also indicate an overall poorer quality of Mol_3 as a substrate for fermentations. This trait might also indicate the low viabilities during the propagation of yeast biomass in this broth (approx. 60% viability for PE-2).

As observed in industrial setups, industrial isolates present a competitive advantage against other *S. cerevisiae* strains (i.e., laboratory adapted), eventually taking over the population along the process by almost completely removing less fit competitors for the industrial process-related stresses. The same trend of removal was observed in the case of the SM and molasses media in the pairwise cultivations, whereas in YPD a larger fraction of the population was still occupied by the YFP-producing laboratory strain.

The synthetic SM medium is able to reproduce sugarcane molasses fermentation with great accuracy, even from different countries. Therefore, this medium could find potential industrial applications, as a tool for media composition optimization, during pre-industrial scales bioprocesses [28, 53, 54, 55], or to more controlled industrial fermentations for the production of high-value commodities [56], by providing optimal growth conditions for any given microorganism, when compared to non-defined complex media, as current industrial substrates. By adjusting its composition one can also consider creating conditions that, besides being optima for the chosen microorganism, are deleterious for potential contaminants [57], potentially increasing maximum fermentation yield and reducing contamination control related costs in large-scale industrial setups.

## Conclusions

In this study, we developed a simple, easily reproducible medium, which efficiently simulated sugarcane molasses-based media. It was successfully used to simulate an industrial bioprocess at the laboratory scale, namely the Brazilian ethanol production process, in order to demonstrate the importance of using industrial relevant media to obtain microbial phenotypes that are closer to industrial conditions.

The development of novel microbial cell factories for future biorefineries requires dealing with the stressful environment found in such bioprocesses [58]. Synthetic media that faithfully simulate industrial conditions and allow for physiological studies regarding its components influence on microbial physiology might play an important role in the selection of novel industrial strains by reducing the time and cost in research and development [27, 59, 60].

## Additional files

**Additional file 1.** The composition of different synthetic molasses tested, and reasons for altering these composition of the first SM tested.

**Additional file 2.** Chemical composition of SM and molasses based media used in this study. Concentrations of total fermentable sugars (sucrose, glucose and fructose), aconitic acid, acetic acid and lactic acid. SM= Synthetic molasses; Mol_1 = molasses 1; Mol_2 = molasses 2; Mol_3 = molasses 3. Values are expressed in g l^−1^.

## Abbreviations

YPD: yeast extract peptone dextrose
LB: Luria broth
MRS: De Man, Rogosa and Sharpe broth
SM: synthetic molasses
Mol_1: molasses 1
Mol_2: molasses 2
Mol_3: molasses 3
PBS: phosphate-buffered saline
OD: optical density
HPLC: high-performance liquid chromatography
ANOVA: analysis of variance
Yse: ethanol yield (g ethanol g substrate^−1^)
Ysc: CO_2_ yield (g CO_2_ g substrate^−1^)
Ysg: glycerol yield (g glycerol g substrate^−1^)
Ysa: acetic acid yield (g acetic acid g substrate^−1^)
NAD: nicotinamide adenine dinucleotide
NADH: nicotinamide adenine dinucleotide hydride
