# Supplementary material for: A synthetic medium to simulate sugarcane molasses
Source: Biotechnol Biofuels. 2018 Aug 11;11:221. doi: 10.1186/s13068-018-1221-x (PMC6086992; doi:10.1186/s13068-018-1221-x)
Supplement: Supplementary file 1 — Additional file 1. The composition of different synthetic molasses tested, and reasons for altering these composition of the first SM tested. [file 13068_2018_1221_MOESM1_ESM.docx]

**Additional File 1:** The composition of different synthetic molasses tested, and reasons for altering these.

**Table S1**: Composition of the first SM tested.

| Category | Nutrient | Concentration |
| --- | --- | --- |
| Carbon sources | Sucrose | 180 |
|  | (NH_4_)_2_SO_4_ | 5 |
|  | (NH_4_)_2_HPO_4_.4H_2_O | 1.42 |
| Nitrogen sources | Trans-aconitic acid | 2 |
|  | L-malic acid | 1 |
|  | Citric acid | 0.01 |
| Organic acids | NaCl | 0.5 |
|  | MgSO_4_.7H_2_O | 1.002 |
|  | CaCl_2_.2H_2_O | 0.06712 |
| Mineral salts | KCl | 0.012 |
|  | MnSO_4_.H_2_O | 0.0004 |
|  | ZnSO_4_.7H_2_O | 0.0004 |
|  | FeCl_3_.6H_2_O | 0.017 |
|  | Na_2_MoO_4_.H_2_O | 0.031 |
|  | KI | 0.012 |
|  | CuSO_4_.5H_2_O | 0.0004 |
|  | H_3_BO_3_ | 0.0005 |
|  | Inositol | 0.01 |
|  | Nicotinic acid | 0.01 |
|  | Calcium pantothenate | 0.001 |
| Vitamins | Biotin | 0.00001 |
|  | Pyridoxine hydrochloride | 0.00004 |
|  | Thiamine hydrochloride | 0.00004 |
|  | Para-aminobenzoic acid | 0.002 |
|  | Ergosterol | 2 ml* |

Sucrose as sole carbon source. (NH_4_)_2_SO_4_ as main nitrogen source.

**Reasons for altering:** Carbon source composition is not as commonly found in sugarcane molasses [1], so it is also for the proportion of organic:inorganic N source [1,2]. *6%, w v^-1^, ergosterol in ethanol solution.

**Table 2:** Composition of second SM tested.

| Category | Nutrient | Concentration |
| --- | --- | --- |
| Carbon sources | Sucrose | 144 |
|  | Glucose | 18 |
|  | Fructose | 18 |
| Nitrogen sources | Peptone | 4.9 |
|  | (NH_4_)_2_SO_4_ | 0.1 |
|  | (NH_4_)_2_HPO_4_.4H_2_O | 1.42 |
| Organic acids | Trans-aconitic acid | 2 |
|  | L-malic acid | 1 |
|  | Citric acid | 0.01 |
| Mineral salts | NaCl | 0.5 |
|  | MgSO_4_.7H_2_O | 1.002 |
|  | CaCl_2_.2H_2_O | 0.06712 |
|  | KCl | 0.012 |
|  | MnSO_4_.H_2_O | 0.0004 |
|  | ZnSO_4_.7H_2_O | 0.0004 |
|  | FeCl_3_.6H_2_O | 0.017 |
|  | Na_2_MoO_4_.H_2_O | 0.031 |
|  | KI | 0.012 |
|  | CuSO_4_.5H_2_O | 0.0004 |
|  | H_3_BO_3_ | 0.0005 |
| Vitamins | Inositol | 0.01 |
|  | Nicotinic acid | 0.01 |
|  | Calcium pantothenate | 0.001 |
|  | Biotin | 0.00001 |
|  | Pyridoxine hydrochloride | 0.00004 |
|  | Thiamine hydrochloride | 0.00004 |
|  | Para-aminobenzoic acid | 0.002 |
|  | Ergosterol | 2 ml* |

The carbon source composition has been altered in order to be more similar to those found in molasses, with the addition of glucose and fructose, in adequate proportions to sucrose [1]. The ratio between organic:inorganic source of nitrogen has also been altered, with the addition of peptone [1,2]. *6%, w v^-1^, ergosterol in ethanol solution.

**Reasons for altering:** This medium lacked Maillard reaction products, from sugar thermal degradation, which are commonly found in molasses and reported to be inhibitory for several microorganisms [3–5].

**Table 3:** Composition of third SM tested.

| Category | Nutrient | Concentration |
| --- | --- | --- |
| Maillard reaction products simulation | Humic acid* | 14.12 |
| Carbon sources | Sucrose | 144 |
|  | Glucose | 18 |
|  | Fructose | 18 |
| Nitrogen sources | Peptone | 4.9 |
|  | (NH_4_)_2_SO_4_ | 0.1 |
|  | (NH_4_)_2_HPO_4_.4H_2_O | 1.42 |
| Organic acids | Trans-aconitic acid | 2 |
|  | L-malic acid | 1 |
|  | Citric acid | 0.01 |
| Mineral salts | NaCl | 0.5 |
|  | MgSO_4_.7H_2_O | 1.002 |
|  | CaCl_2_.2H_2_O | 0.06712 |
|  | KCl | 0.012 |
|  | MnSO_4_.H_2_O | 0.0004 |
|  | ZnSO_4_.7H_2_O | 0.0004 |
|  | FeCl_3_.6H_2_O | 0.017 |
|  | Na_2_MoO_4_.H_2_O | 0.031 |
|  | KI | 0.012 |
|  | CuSO_4_.5H_2_O | 0.0004 |
|  | H_3_BO_3_ | 0.0005 |
| Vitamins | Inositol | 0.01 |
|  | Nicotinic acid | 0.01 |
|  | Calcium pantothenate | 0.001 |
|  | Biotin | 0.00001 |
|  | Pyridoxine hydrochloride | 0.00004 |
|  | Thiamine hydrochloride | 0.00004 |
|  | Para-aminobenzoic acid | 0.002 |
|  | Ergosterol | 2 ml** |

Humic acid was added as a new component, in order to simulate the Maillard reaction products [4], generated during the molasses production process. *Not a nutrient. **6%, w v^-1^, ergosterol in ethanol solution.

**Reasons for altering:** Humic acid substances have electric charges, causing it to bind to microbial cells during fermentation. When centrifuging to quantify microbial biomass production, most of the humic acid is removed as well, making it impossible to correctly quantify biomass.

**Table 4:** Composition of fourth and final SM tested.

| Category | Nutrient | Concentration |
| --- | --- | --- |
| Maillard reaction products simulation | Glutamine | 19 |
|  | Aspartic acid | 11 |
|  | Asparagine | 7.1 |
| Carbon sources | Sucrose | 144 |
|  | Glucose | 18 |
|  | Fructose | 18 |
| Nitrogen sources | Peptone | 4.9 |
|  | (NH_4_)_2_SO_4_ | 0.1 |
|  | (NH_4_)_2_HPO_4_.4H_2_O | 1.42 |
| Organic acids | Trans-aconitic acid | 2 |
|  | L-malic acid | 1 |
|  | Citric acid | 0.01 |
| Mineral salts | NaCl | 0.5 |
|  | MgSO_4_.7H_2_O | 1.002 |
|  | CaCl_2_.2H_2_O | 0.06712 |
|  | KCl | 0.012 |
|  | MnSO_4_.H_2_O | 0.0004 |
|  | ZnSO_4_.7H_2_O | 0.0004 |
|  | FeCl_3_.6H_2_O | 0.017 |
|  | Na_2_MoO_4_.H_2_O | 0.031 |
|  | KI | 0.012 |
|  | CuSO_4_.5H_2_O | 0.0004 |
|  | H_3_BO_3_ | 0.0005 |
| Vitamins | Inositol | 0.01 |
|  | Nicotinic acid | 0.01 |
|  | Calcium pantothenate | 0.001 |
|  | Biotin | 0.00001 |
|  | Pyridoxine hydrochloride | 0.00004 |
|  | Thiamine hydrochloride | 0.00004 |
|  | Para-aminobenzoic acid | 0.002 |
|  | Ergosterol | 2 ml** |

The simulation of the Maillard reactions originating from the molasses production processes was achieved by preparing a concentrated stock sugar solution (l^-1^: sucrose (432 g); glucose (54 g); and fructose (54 g)). In this stock solution, the most common amino acids normally found in sugarcane juice were added as follows [1]: (l^-1^) glutamine (57 g); aspartic acid (33 g); and asparagine (21,3 g). This solution was then autoclaved at 121°C for 15 min (“liquids” program, 2 bar pressure, CertoClave Multicontrol. Certoclave, Traun, Austria) [6,7].

This medium was later benchmarked regarding yeast fitness and growth, as also its fermentability against different actual molasses, proving to be a faithful simulation.

**References**

1. Olbrich H. The molasses [Internet]. Biotechnol. Kempe GmbH. Biotechnologie-Kempe GmbH; 1963. Available from: http://www.biotechnologie-kempe.de/Molasses_OLBRICH.pdf

2. Hashizume T, Higa S, Sasaki Y, Yamazaki H, Iwamura H, Matsuda H. Constituents of Cane Molasses Part 1. Separation and Identification of the Nucleic Acid Derivatives. Agric Biol Chem BioI Chern [Internet]. 1966 [cited 2018 Mar 13];304:319–29. Available from: http://www.tandfonline.com/action/journalInformation?journalCode=tbbb19

3. Kumar P, Chandra R. Decolourisation and detoxification of synthetic molasses melanoidins by individual and mixed cultures of Bacillus spp. Bioresour Technol [Internet]. 2006 [cited 2018 Jan 24];97:2096–102. Available from: http://production.datastore.cvt.dk/filestore?oid=539cf8ba5c6bfcd1250267ed&targetid=539cf8ba5c6bfcd1250267ef

4. Hatano K, Kikuchi S, Miyakawa T, Tanokura M, Kubota K. Separation and characterization of the colored material from sugarcane molasses. Chemosphere [Internet]. 2008;71:1730–7. Available from: http://linkinghub.elsevier.com/retrieve/pii/S0045653507015445

5. Chandra R, Bharagava RN, Rai V. Melanoidins as major colourant in sugarcane molasses based distillery effluent and its degradation. Bioresour Technol [Internet]. 2008 [cited 2018 Jan 24];99:4648–60. Available from: http://production.datastore.cvt.dk/filestore?oid=539d2ab81e2dbdd325046515&targetid=539d2ab81e2dbdd325046517

6. Golon A, Kropf C, Vockenroth I, Kuhnert N. An Investigation of the Complexity of Maillard Reaction Product Profiles from the Thermal Reaction of Amino Acids with Sucrose Using High Resolution Mass Spectrometry. Foods [Internet]. 2014;3:461–75. Available from: http://www.mdpi.com/2304-8158/3/3/461/

7. Ames JM, Wynne a, Hofmann a, Plos S, Gibson GR. The effect of a model melanoidin mixture on faecal bacterial populations in vitro. Br J Nutr [Internet]. 1999;82:489–95. Available from: http://www.ncbi.nlm.nih.gov/pubmed/10690164
