# Supplementary material for: A synthetic medium to simulate sugarcane molasses
Source: Biotechnol Biofuels. 2018 Aug 11;11:221. doi: 10.1186/s13068-018-1221-x (PMC6086992; doi:10.1186/s13068-018-1221-x)
Supplement: Supplementary file 2 — Additional file 2. Chemical composition of SM and molasses based media used in this study. Concentrations of total fermentable sugars (sucrose, glucose and fructose), aconitic acid, acetic acid and lactic acid. SM= Synthetic molasses; Mol_1 = molasses 1; Mol_2 = molasses 2; Mol_3 = molasses 3. Values are expressed in g l−1. [file 13068_2018_1221_MOESM2_ESM.docx]

| Component/Broth | SM | Mol_1 | Mol_2 | Mol_3 |
| --- | --- | --- | --- | --- |
| Total sugars | 180.00±4.48 | 188.02±2.81 | 165.31±1.86 | 131.36±2.65 |
| Aconitic acid | 2.13±0.25 | 1.81±0.02 | 1.98±0.04 | 0.66±0.02 |
| Acetic Acid | 0.88±0.43 | 0.42±0.09 | 0.76±0.45 | 1.12±0.59 |
| Lactic Acid | 0.02±0.01 | 1.80±0.07 | 2.63±0.08 | 1.86±0.02 |

**Additional File 2**: Chemical composition of the media used in this study. Concentrations of total fermentable sugars (sucrose, glucose and fructose), aconitic acid, acetic acid and lactic acid. SM= Synthetic molasses; Mol_1 = molasses 1; Mol_2= molasses 2; Mol_3 = molasses 3. Values are expressed in g l^-1^.
